# Understanding Indigenous Exploitation Through Performance Based Research Funding Reviews in Colonial States

**DOI:** 10.3389/frma.2020.563330

**Published:** 2020-11-11

**Authors:** Tyron R. Love, C. Michael Hall

**Affiliations:** ^1^ Management and International Business, University of Auckland, Auckland, New Zealand; ^2^ Management, Marketing and Entrepreneurship, University of Canterbury, Christchurch, New Zealand; ^3^ Geography, University of Oulu, Oulu, Finland; ^4^ Service Management and Service Science, Lund University, Helsingborg, Sweden; ^5^ Tourism, School of Business and Economics, Linnaeus University, Kalmar, Sweden

**Keywords:** indigenous research, indigenous knowledge, Māori episteme, indigenous exclusion, tokenism, economization, performance based research Fund, neoliberalism

## Abstract

Countries with significant indigenous populations, such as Australia, New Zealand and the Nordic countries, are providing increased support for improvements in the number of indigenous academics represented in higher education and engaged in research. Such developments have occurred at the same time as the implementation of performance-based research funding systems. However, despite the significance of such systems for academic careers and knowledge diffusion there has been relatively little consideration of the way within which they meet the needs of indigenous academics and knowledges. Drawing primarily on the New Zealand context, this perspective paper questions the positioning of Māori researchers and Māori research epistemologies (Kaupapa Maori) within the Performance Based Research Fund and the contemporary neoliberal higher education system. It is argued that the present system, rather than being genuinely inclusive, serves to reinforce the othering of Māori episteme and therefore perpetuates the hegemony of Western and colonial epistemologies and research structures. As such, there is a need to raise fundamental questions about the present ecologies of knowledge that performance based research systems create not only in the New Zealand higher education research context but also within other countries that seek to advance indigenous research.

## Introduction

For more than 50 years, indigenous scholars have published research as agents of researching entities (universities, colleges, institutes) in colonised nations. More than that, those entities have created schools, institutes and programmes specifically dedicated to produce research outputs which recognise the distinct political and intellectual traditions of indigenous peoples (Simpson and Smith, 2014). At one level such actions are framed by government and higher education policies as contributing to indigenous knowledge so as to improve the often parlous socio-economic, health and environmental circumstances of indigenous peoples (Buckskin et al., 2018). Often these initiatives can be seen as governmental and other political and economic responses to the political actions of the first organized modern indigenous movements (Minde et al., 2008) in modern colonial states; including the First Nations, Métis and Inuit peoples of Canada; the Native American peoples of the United States; the Māori people of New Zealand; the Aboriginal and Torres Strait Islander peoples of Australia; and the Sami peoples of Norway, Finland, Sweden, and Russia. However, the incorporation of indigenous knowledge and the research of indigenous scholars into the “industrial actor-network of academic knowledge production, circulation and reception” (Gibson and Klocker, 2004: 425) raises fundamental questions of not only ownership of indigenous knowledge but also its acceptability, assessment, encouragement, ethics and representation in research quality evaluations and metrics (PBRF Review Panel, 2020). The present paper offers a perspective on these issues.

## Indigenous Knowledges and Their Assessment

In seeking to understand indigenous scholarly research there is no ‘‘view from nowhere’’ since knowledge is always ‘‘local, situated and embedded’’ (Shapin, 1998: 6). Contemporary indigenous ways of researching demand that researchers are accountable (Bainbridge et al., 2015), requiring them to consider what their researching hopes to achieve for people and the communities being researched (Durie, 2017; PBRF Review Panel, 2020). Researchers are obliged to create and maintain credibility and integrity when working within Indigenous communities; research cannot be about “simply rushing in and ‘ripping off’ communities in order to obtain personal credentials” (Smith, 1997: p. 93). This guidance stems from a history of research which has been detrimental in many respects to Indigenous peoples (Smith, 2012). Recognition of how knowledge is produced, circulated and assessed is therefore fundamental to establishing its credibility, its beneficiaries and how it is read in different places (Hall, 2013). It is at the local level that research gets its audience and therefore, “its motivation, relevance, application and strength” (Roa et al., 2009: 234).

Indigenous knowledges are each reflected in their relatedness to specific milieu, culture and place but are becoming increasingly circulated and diffused within institutionalized national and international assessment systems that serve to assess the worthiness of indigenous knowledge as a world-view and as a subject for publication. Nevertheless, institutional norms and practices of Western universities often conflict with indigenous ways of researching (Rios et al., 2020). This is witnessed in various performance-based reward systems which have indigenous subjects within them, such as the Performance-based Research Funding Systems in Denmark, Sweden, Norway and Finland (Söderlind et al., 2019); Australia’s Excellence in Research for Australia (ERA) exercise (Buckskin et al., 2018; Woelert and McKenzie, 2018), and New Zealand’s Performance Based Research Funding (PBRF) programme (Roa et al., 2009; PBRF Review Panel, 2020). However, to this could also be added the various forms of impact and quality assessments of individual publications, including as to whether publications are included in the “right” bibliometric database or not (Hobson and Hall, 2010), and the related development of university and institutional rankings (Kauppi, 2018). Such systems of surveillance often have direct impacts on how and on what research is conducted and where it is published (Hall, 2005), with corresponding sets of incentives and disincentives to do so which affect not only individual research careers but also the capacity to voice different forms of indigenous (and gendered and localized) knowledge and research practices (Hall, 2013). From an indigenous perspective, such a situation may only further reflect colonial knowledge relationships as the various institutional arrangements for assessing knowledge will frame the local knowledge of the colonised in relation to the knowledge and assessments of the coloniser (Connell, 2007; Smith, 1999, Smith, 2012). Indeed, in the case of the ERA it has been recognised that although the assessment of research quality using a combination of metrics focused on researchers, research outputs, research income, esteem and applied measures is quantifiable, it does not generally translate into measures valued by research end-users including, in the case of health research, indigenous Australians that “have been over-researched without corresponding improvements in their health” (Bainbridge et al., 2015).

The phenomenological relationship between the geographical contexts of being somewhere and knowledge acquisition reflects a concern not just with where things matter but also how and why they matter. We write from the perspective of academics currently working in New Zealand who have spent time in New Zealand, Australia, Canadian and Nordic universities. We have indigenous (TL) and non-indigenous (CMH, TL) ancestry and are engaged with indigenous and non-indigenous knowledges not only in terms of research but also research administration. Our concerns with indigenous knowledge assessment come therefore from seeking to understand how indigenous knowledges and research becomes incorporated in practices of consumption, production and marketization of the global circulation of knowledge in which it is assessed and subsequently reframed and returned to the local as part of new practices, for example the “world class” (Mittelman, 2017) and “entrepreneurial university” (Deem, 2001), accreditation schemes, and the incorporation of cultural “competence” into higher education in order to “tick off the box” that students have acknowledged indigenous populations (Carey, 2015). Indeed, the notion of cultural “competence” has been substantially criticized, being regarded by some as a perpetuation of a racist knowledge paradigm (Pon, 2009) because of the failure of broader social and economic systems to acknowledge indigenous world views (de Leeuw, 2019).

### Liberal Exclusion of the Indigenous

A history of liberal exclusion through processes of colonisation have brought about a sad and enduring period in indigenous-colonial relations. The first universities in colonised states were built on a solid foundation of liberal philosophy whereby universities sought their intellectual freedom and autonomy from political, social, and economic institutional forms. Simultaneously, they sought independence from long-standing indigenous institution intellectualisms, customs, knowledges, and languages; solid institutional forms in their own right. There was no room for indigenous theory, methodologies, analyses in the academy (Walker, 2016). This is far from a history which has ended. Forms of indigenous exclusion still exist (McAllister et al., 2019). Potentially crucially for performance based funding reviews, “In the same way that indigenous peoples and their epistemes remained invisible when the nation-states were being shaped, indigenous scholarship remains invisible and unreflected in most academic discourses” (Kuokkanen, 2011: 156).

The underrepresentation of indigenous researchers in Western universities and other higher education institutes has serious consequences for building research environments which foster feelings of inclusion and for advancing research programmes where collegiality is operationalised as opposed to simply being a line in a policy document. In New Zealand, indigenous Māori faculty make up only 6% of the university academic workforce despite being 14.9% of the population in general (Kidman and Chu, 2015; McAllister et al., 2019). The situation is equally disturbing in the United States where Native American peoples make up only 0.5% of the academic faculty in higher education despite comprising roughly 2% of the population (Walters et al. 2019). Likely the product of adverse academic experiences for indigenous peoples, such underrepresentation is unsurprising given the inequities faced by indigenous faculty who often have a different occupation shape and career trajectory to their peers (Middleton and McKinley, 2010), are more likely to witness or experience discrimination (Henry et al., 2017), are much less likely to be on permanent contracts, and who have much shorter careers than other researchers in the ethnic majority (Kidman and Chu, 2015), who experience hurtful public censures from non-indigenous academics (Calhoun, 2003), and who face identity related tokenism (e.g., increased scrutiny, stereotyping) and exclusion (feeling invisible, lack of belonging) (Henry et al., 2017; McAllister et al., 2019; Naepi, 2019; Settles et al., 2019). Such feelings and experiences can have devastating impacts on indigenous faculty trying to prove their worth in colonial institutions pushing neoliberal agendas of excellence. As such, indigenous faculty “report intellectual and emotional isolation and loneliness in research institutions” (Brayboy et al., 2012, in Walters et al., 2019: 613), face situations “loaded with the deck stacked against them” (Fenelon, 2003: 90), and have their identities questioned when they straddle two or more worlds (Hernández-Avila, 2003).

## MĀori and the Performance Based Research Fund

The New Zealand PBRF government initiative, first run in 2003 to assess the quality of academic research in degree-granting organisations, has been blamed for creating barriers to research for indigenous Māori academics and associated methodologies and worldviews (Roa et al. 2009; PBRF Review Panel, 2020) and, in turn, causing a decrease in the numbers of Māori researching academics. Prominent indigenous professor, Linda Tuhiwai Smith made the observation that in 2003, 599 indigenous Māori academics submitted research portfolios to the PBRF and by 2012 that number had halved to 290 (Smith, 2016). The most recent results in 2018 also reveal “a lag in the number of Māori… researchers participating in the PBRF.” (Tertiary Education Commission/Te Amorangi Mātauranga Matua (TEC), 2019a: 36). The PBRF assessment has led to a strong focus on various metrics such as the number and quality of publications produced by academics. The significance of numbers and relative subject ranking is also reflected in the promotion by universities of how they have performed in the PBRF exercise. However, as Kauppi (2018) has recognized, as a highly objectified form of knowledge, data has a material force of its own, and gives the impression of providing access to a more profound level of reality and subsequently “it influences, with varying impact, actors’ behavioral patterns”.

### Neoliberalism and the Economization of the PBRF

The development of performance assessments, such as PBRF, reflects that New Zealand higher education has entered a period of neoliberal exploitation which, far from succeeding the liberal tradition, has simply added to pre-existing university managerialism. A result of which is that universities have been increasingly driven by a corporate business logic enabled through market mechanisms (Roberts, 2014: 12) obsessed with achieving diversity quotas and crafting equity agendas that can be included in government reporting**.** In the case of the development of performance based funding this has led to a process of economization—the assembly of actions, behaviors, devices, institutions, objects and analytical/practical descriptions which are tentatively and sometime controversially qualified as ‘economic’ by scholars, lay people, government actors and/or market actors (Çalıskan and Callon, 2009; Hall, 2011). In the case of the PBRF such measures are primarily assessed at the level of the submitted individual performance, with the results later amalgamated for the purpose of institutional funding; a utilitarian action and framing of the research process, which in itself is in opposition to indigenous notions of the collective and communitarian nature of research (Denzin, 2008). Nevertheless, the PBRF sets the rules of the research game in New Zealand (PBRF Review Panel, 2020). The implications of which are driven down to the individual career level via human resource management and reward systems. The imposition of the rules of the game, “the rules used to calculate decisions”, as Callon (1998: 46) observed, are used as “the starting point of relationships of domination which allow certain calculating agencies to decide the location and distribution of surpluses. The extension of a certain form of organized market, an extension which ensures the domination of agents who calculate according to the prevailing rules of that particular market, always corresponds to the imposition of certain calculating tools.”

In New Zealand the early neoliberalism of the 1980s sought to reduce the role of government in the management of public sector institutions but was later replaced by government efforts to remodel the sector on neo-liberal, free-market private-sector management principles (Shore, 2010). As a result, with the 1990s came significant changes to the higher education sector recognising indigenous tertiary institutions (wānanga) making them eligible for state funding, the number of indigenous students graduating with tertiary degrees increased, and university charters were required to show how they were committing to the principles of New Zealand’s founding document, the *Treaty of Waitangi* (Durie, 2009). This has meant that in the era of “post-colonialism” governments are relatively proactive at creating indigenous committees or appointing an indigenous academic to a panel to consider the fit. Indigenous peoples have created academic careers in the era of the neoliberal university (Kidman and Chu, 2017), but, as a result of legislative and policy changes, the “inclusion” of indigenous faculty in processes to create, monitor and change the rules of assessing and promoting research performance are mostly tokenistic and performative (Pidgeon, 2016; Henry et al., 2017; Staniland, 2017). Under the PBRF, for example, there is a specific panel to address Māori Knowledge and Development which awarded funding to 174.87 Evidence Portfolios (EPs) (FTE weighted) in 2018 as compared to 125.83 EPs in 2012. Māori researchers comprised 88.6% of those submitting to the MKD Panel in 2018, an increase from 74.5% in 2012 although, significantly for Māori research in the longer term, only 15.5% of researchers who submitted to MKP were aged under 40 (TEC, 2019b).

### Empowering or Othering?

From some perspectives the establishment of a separate panel to asses Māori research within the PBRF may be regarded as a positive (PBRF Review Panel, 2020). However, notions of empowerment can sometimes be more patronising than helpful and may not lead to any real transformation (Kuokkanen, 2011). Universities and the government’s funding of excellence are caught up in the pursuit of being seen to address inequities and improve the numbers. But instead, just focusing on numbers arguably only serves to reinforce colonial logic. Māori research is put in a separate box—perhaps with the best of intentions—but such an approach does not really assist in “the development of power sharing processes, and the legitimation of diverse cultural epistemologies and cosmologies” (Bishop, 2006: 3). Instead, such binary practices may “perpetuate the ideology of cultural superiority that is fundamental to colonisation” (Bishop, 2006: 3) by framing Māori research epistemologies (Kaupapa Maori) as other (Cooper, 2012) in a manner that is, arguably, symptomatic of contemporary academic colonialism. Rather than empowering indigenous research voices to engage in a conversation with the wider academy and its epistemologies, the processes of academic surveillance and control that the PBRF represents, instead becomes a site for the continued dominance of Western world-views in the New Zealand academy and reproduces the current epistemological hegemony.

Tawhai et al. (2004) in their critical reflections on the establishment of the PBRF system and, in particular ,the role of the government in determining its nature, highlight the poor levels of consultation with indigenous Māori which led to a system partially ignorant of indigenous research processes in its early stages. For example, consider the act of creating *individual* research portfolios and putting them forward for research quality evaluation. From an indigenous Māori perspective, such a task requires a considerable psychological character shift from one rooted primarily in a history of humility and modesty to one of arrogance and showing off (Tawhai et al., 2004). This is a salient issue often not recognized by non-indigenous scholars and assessment systems, with respect to what is being measured. Indigenous scholars who are lauded locally as carvers, weavers, performers and storytellers and who have learnt skills passed down over generations feel aggrieved to claim their work as their own, as in the case of PBRF evidence portfolio purposes, when their efforts and labor are to them absolutely inseparable from that of their ancestors, teachers, and elders regardless of the disciplinary panel into which they are categorized (Bishop, 2006, Bishop, 2011).

## Conclusion

There is an urgent need for research performance based funding systems and their implications to be rethought from an indigenous perspective. The utilitarian model of such performance models do not meet the needs of indigenous researchers and their communities and those working within indigenous epistemologies, although it does meet the needs of the neoliberal university and higher education research governance. In a health context there has been substantial recognition of the need for new approaches to indigenous health, wellbeing and research. In New Zealand, Australia and Canada the notion of “cultural safety” has been promoted as a way of “shifting from deficit-based discourses and the associated individualizing of health concerns toward a recognition that larger processes of colonization, including the loss or attenuation of traditional beliefs, practices and language, need to be taken into account in any explanation of, and attempt to improve, health for Indigenous peoples” (de Leeuw, 2019). Similarly, in a Southern higher education and epistemological context, Santos (2018) calls for “new kinds of theoretical, epistemological, organization, and pedagogical orientations,” as part of the development of new ecologies of knowledge. What is now needed in the assessment of indigenous knowledge via performance based funding reviews is the development of a research evaluative concern with cultural safety that better recognizes not only indigenous worldviews and languages but also research practices, including forms of knowledge transfer and assessment that do not conform with some of the standard models of journal publishing and peer review, but which are no less relevant or valuable (Smith, 2017). This will require a framing of the value of indigenous episteme across the entire process and not just being placed in a separate category of research that only serves to reinforce otherness.

Bainbridge et al. (2015) in commenting on the “elephant in the room” in terms of issues of research impact and benefit in Aboriginal and Torres Strait Islander health research observed that, “The research impact debate must take account of the various standards of accountability (to whom), impact priorities (for whom), positive and negative impacts, and biases that operate in describing impact and measuring benefit”. We certainly agree with these assessments in the wider indigenous research assessment context but we would also add issues of who is doing the research? (is partnership acknowledged), how it is being undertaken? (is due recognition being given to indigenous knowledge paradigms and research practices), and how it is being communicated? (is knowledge communication appropriate in indigenous terms). In addition, we suggest that there is an urgent need for greater recognition of the consequences of research performance reviews on the careers of indigenous staff at higher education institutions given their current Western colonial bias with respect to notions of research excellence.

## Data Availability Statement

The original contributions presented in the study are included in the article/supplementary material, further inquiries can be directed to the corresponding author.

## Author Contributions

Both authors contributed equally to the paper.

## Conflict of Interest

The authors declare that the research was conducted in the absence of any commercial or financial relationships that could be construed as a potential conflict of interest.

## Funding

This research is part of the Marsden funded research project “It looks grim: The future of Māori academics in New Zealand universities” (Project ID: 19-UOC-005).
